# Complementary Strategies for Temporal Lifting Techniques: Biomechanical Insights From Combined Injection Techniques

**DOI:** 10.1111/jocd.70500

**Published:** 2025-11-04

**Authors:** Wencheng Fan, Yanping Gao, Xiaohui Qin, Lin Chen, Li Zou, Chuanxiu Zeng, Linghuan Zeng

**Affiliations:** ^1^ Department of Aesthetic Medicine Sichuan Integrative Medicine Hospital Chengdu Sichuan China; ^2^ Tanmei Medical Aesthetic Clinic Wuhou, Chengdu Sichuan China

## Abstract

**Background:**

Although the use of hyaluronic acid–based soft tissue fillers in the lateral facial region is known to have full‐face aesthetic effects, systematic comparisons of injection techniques remain limited.

**Aims:**

To compare the full‐face effects of posterotemporal lifting alone and combined with supraperiosteal zygomatic arch injection, and to evaluate the use of images from three device types for aesthetic assessment.

**Methods:**

Data from 20 female Asian patients (group A, 38.0 ± 6.29 years; group B, 41.5 ± 6.72 years) was analyzed retrospectively. Group A received single‐point subcutaneous posterosuperior temporal injections, and group B additionally received supraperiosteal zygomatic arch injections. Outcomes were assessed using the Global Aesthetic Improvement Scale (GAIS) and three‐dimensional (3D) quantifications of volume and vector changes in the medial (maxillary and medial mandibular regions) and lateral [temporal region (superolateral face) and lateral mandibular region (inferolateral face)] face. GAIS ratings of images obtained with three device types were compared.

**Results:**

Mean local (superolateral, inferolateral, and medial face) surface volume changes were 0.46 ± 0.36, 1.16 ± 1.03, and 0.03 ± 0.55 cubic centimeter (cm^3^), respectively, in group A, and 0.23 ± 0.38, −0.76 ± 0.96, and −0.37 ± 0.60 cm^3^, respectively, in group B. More vector displacement in the inferolateral and medial face was generally observed in group B. GAIS scores favored group B over group A, and 3D imaging over two‐dimensional imaging.

**Conclusion:**

Combined temporal lifting with supraperiosteal zygomatic arch injection may achieve better facial lifting effects than simple temporal lifting performed with the same filler volume.

## 1 Introduction

Facial aging is caused by the combined actions of multiple factors. It is not limited to the reduction of facial bone and soft tissue volumes, but also encompasses changes in the skin, muscles, and fascia [1, 2, 3, 4, 5, 6]. Soft tissue fillers address facial aging by restoring volume and lifting soft tissues, with efficacy tied to facial anatomical characteristics. They are applied with consideration of the anatomical characteristics of different facial regions. Four ligaments in the face (the temporal ligament adhesion, lateral orbital thickening, and zygomatic and mandibular ligaments) form a line delineating medial (expression) and lateral (masticatory function) regions [7]. The expression region generally has an “overlapping‐tile” structure. For example, the levator labii superioris alaeque nasi and zygomaticus minor muscles cover part of the levator labii superioris muscle, and these muscles together cover the levator anguli oris muscle. The depressor anguli oris muscle covers part of the depressor labii inferioris muscle, which in turn partially covers the mentalis muscle. The masticatory function region has a parallel anatomical structure with distinct layers and clear boundaries [8]. Shallower injections are associated with weaker superficial tissue restriction of the filler, more local tissue bulging, and stronger lifting efficiency, whereas deeper injections involve more layers and a greater thickness of superficial tissue and restriction by the dense deep fascia, and are associated with less local tissue bulging and weaker lifting efficiency. This phenomenon is named the “onionskin‐like effect,” with reference to the structure of the lateral facial region [8]. Filler injection into this region can reduce nasolabial folds, marionette lines in the middle face, and skin sagging along the mandibular line to various degrees [9].

**FIGURE 1:**
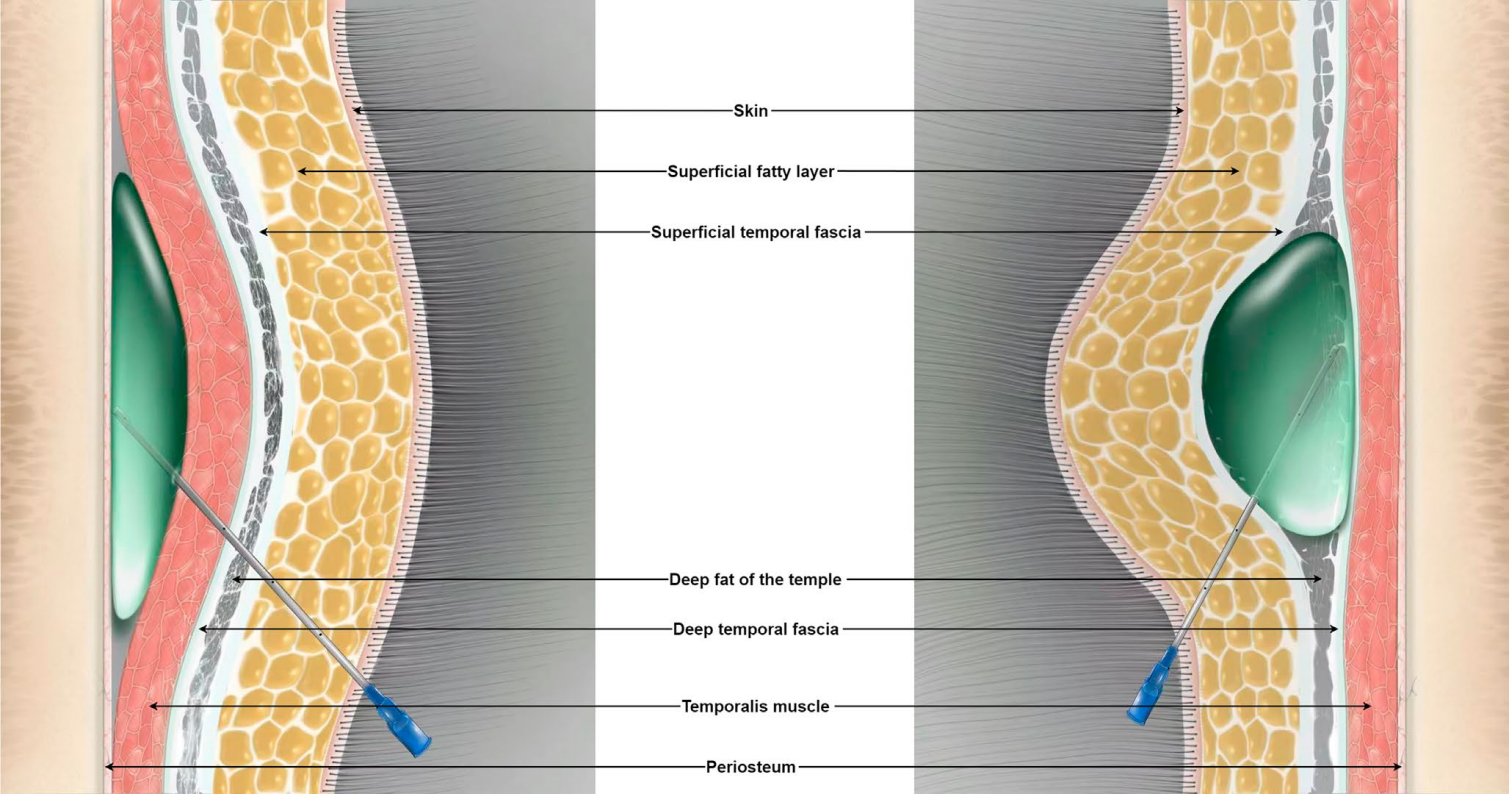
Schematic illustration of local tissue bulge induced by filler injections at different temporal layer depths.

The temporal region is among the filler injection sites in the lateral facial region that is most likely to produce a lifting effect outside of the filling area [10]. Shallow injection (into the superficial fatty layer or superficial aspect of the deep temporal fascia) in this region can have a better lifting effect in the lateral mandibular region (inferolateral face) than deep injection; deep injection (into the deep aspect of the deep temporal fascia, the temporalis muscle, or the supraperiosteal layer) is restricted by the density of the deep temporal fascia and results in minimal bulging and weak lifting efficiency (Figure 1) [11]. In clinical practice, we have also found that the mechanical conduction of the facial skin and soft tissue displacement caused by superficial bulging are attenuated significantly when the “line of ligaments” is crossed, potentially because the ligaments are denser and less extensible than the skin. Although this dense structure restricts soft tissue sagging, it may also limit tissue lifting. For example, when performing facelift surgery, subcutaneous dissection that crosses the ligament line anteriorly to reach the area above the nasolabial groove has a significantly better effect than dissection that does not cross the line [7, 12]. To verify this apparent difference in full‐face lifting effects, we analyzed the differences between the two injection protocols using a combination of subjective evaluation and objective index analysis.

## 2 Material and Methods

### 2.1 Sample

Data from the medical records of patients who underwent temporal facelifts using the hyaluronic acid–based soft tissue filler Persnica (Hugel Inc., South Korea) were reviewed retrospectively. The inclusion criteria were: age 25–55 years, body mass index (BMI) 18.5–24.8 kg/m^2^, availability of complete and standardized preoperative and 1‐month postoperative imaging data, and self‐evaluation of aesthetic changes using the 5‐point Global Aesthetic Improvement Scale (GAIS) in the first postoperative month. Stratified random sampling from two predefined cohorts [patients receiving single‐point subcutaneous injection into the posterosuperior temporal region alone (cohort A) and with supraperiosteal injection into the zygomatic arch (cohort B)] using computer‐generated random numbers resulted in the selection of 10 female Asian patients from each cohort. Any residual baseline differences in age or BMI were adjusted for in multivariable models. Exclusion criteria were: severe skin laxity, a history of allergy to sodium hyaluronate or anesthetic drugs, severe skin infection at the injection site, a history of orthodontic treatment or maxillofacial plastic surgery, a history of thread lift treatment or soft tissue filler injection, congenital or pathological severe exophthalmos, and any pathological condition affecting the facial morphology and expression. All participants were informed of the study's purpose and scope and provided explicit written consent to the use of their de‐identified data and photographs for research, educational purposes, and subsequent publication of this manuscript. This study was conducted in strict compliance with the ethical principles outlined in the Declaration of Helsinki and other relevant international guidelines.

### 2.2 Procedure

**FIGURE 2:**
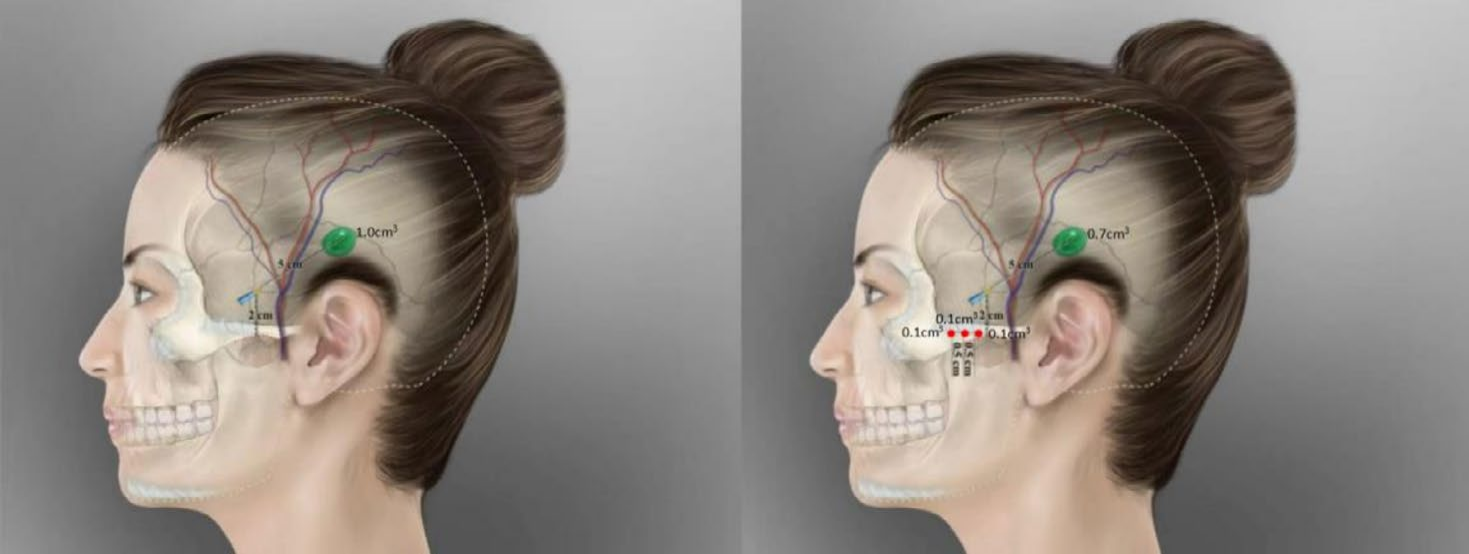
Schematic illustration of two injection protocols: Group A (left) and Group B (right). Yellow circles: Entry points of blunt cannula; Red circles: Entry points of sharp needle and filler injection sites (0.1 cm^3^ per site); Schematic filler depots (Group A: 1.0 cm^3^; Group B: 0.7 cm^3^): Filler deposition sites via blunt cannula.

Before performing the procedure, the physician interviewed each patient and comprehensively evaluated the injection risks. The needle entry point was set 2 cm above the midpoint of the lower edge of the zygomatic arch. For local anesthesia, 0.1 cm^3^ 2% lidocaine was injected into this point. A small, disposable, sterile acupotomy (0.50 × 50 mm) was used to make a hole, through which a 23G blunt cannula (0.60 × 50 mm) was inserted and advanced posterosuperiorly in the lifting direction (at a 45°–60° angle from horizontal) along the superficial layer of the deep temporal fascia until reaching the hair‐bearing region adjacent to the supra‐auricular area. Patients in group A received single‐point injections of 1.0 cm^3^ filler at this site (one each on the left and right sides). Patients in group B received single‐point injections of 0.7 cm^3^ filler at the same basic site (one each on the left and right sides). With this technique, the blunt cannula is directed at an angle, rather than being parallel to the frontal and parietal branches of the superficial temporal artery and vein and the superficial and deep branches of the middle temporal artery and their accompanying veins, reducing the likelihood that it will puncture the blood vessels and cause embolism. Patients in group B received a 0.1‐cm^3^ filler injection with a sharp needle at the junction of the zygomatic process of the temporal bone and the temporal process of the zygomatic bone (zygomatic arch) on each side, with the needle advanced in the supraperiosteal plane. Additional 0.1‐cm^3^ injections on each side were performed with a sharp needle in the supraperiosteal plane 0.5 cm anterior and 0.5 cm posterior to the reference point with a thin‐walled, sharp 25G (13‐mm) needle. Aspiration was performed carefully, and the procedures were carried out gently to avoid damaging blood vessels and causing complications such as hematoma or embolism (Figure 2). All facial injection protocols mentioned in this study are based on the core principle of “bilateral symmetric operation,” and asymmetric facial injection is clearly not advised.

### 2.3 Image Acquisition and Processing

**FIGURE 3:**
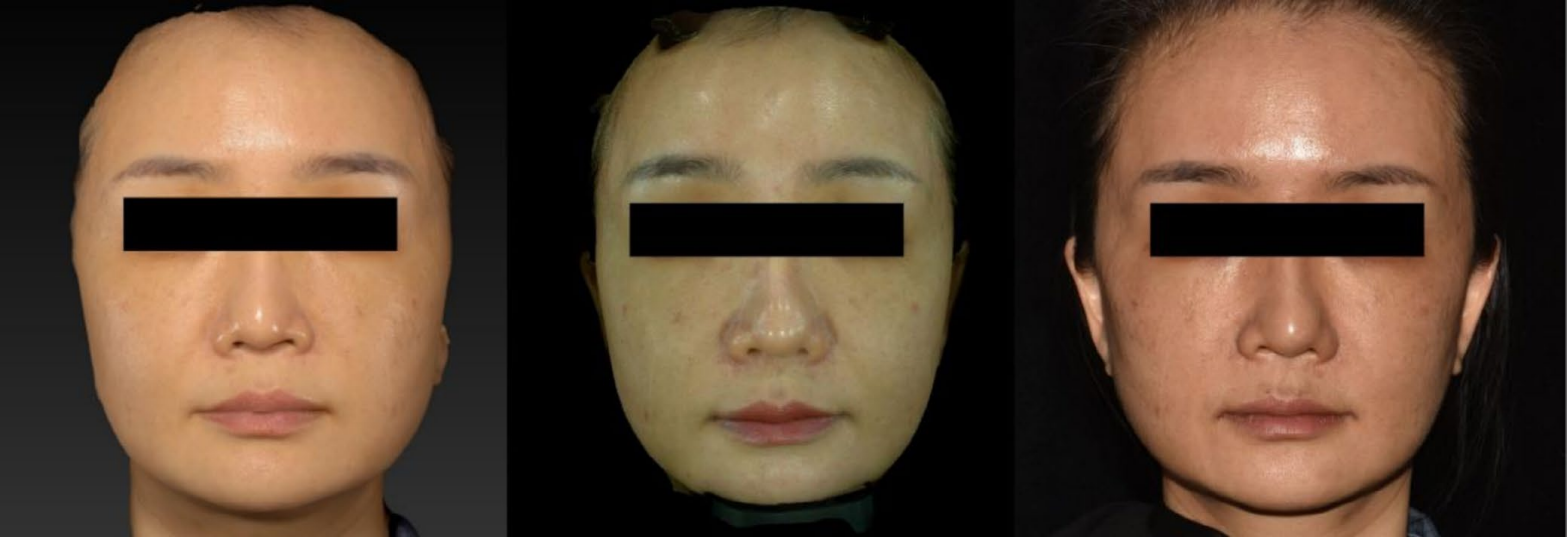
A female patient underwent imaging using three different devices prior to treatment, captured in frontal view: VECTRA H2 (left), MIRAGE P2 (center), and Nikon D750 (right).

The same imaging standards and procedures were used for all patients. Before treatment and during all follow‐up visits, a digital camera (D750; Nikon Inc., Melville, NY, USA) was used to capture facial photographs, including frontal, bilateral 45° oblique, and bilateral 90° lateral views with the head in the Frankfort horizontal plane. A Vectra H2 imaging system (Canfield Scientific Inc., Parsippany, NJ, USA) and a Mirage P2 3D Smart Skin Analyzer (Xiaofu Technology Co. Ltd., Hangzhou, China) were used to acquire and synthesize three‐dimensional (3D) images (Figure 3).

### 2.4 Clinical Evaluation

Three ordinary observers were recruited to evaluate the pretreatment and 1‐month posttreatment images. They viewed the 3D images obtained with the Vectra H2 and Mirage P2 systems and the multi‐angle digital photographs. The observers were able to adjust the 3D images to any angle. The observation time for each image set was 1 min. The observers used the GAIS to rate the aesthetic results of the intervention relative to baseline [1, worse (the appearance is worse than the original condition); 2, no change (the appearance is essentially the same as the original condition); 3, improved (obvious improvement in appearance from the initial condition, but touch‐up or retreatment is indicated); 4, much improved (marked improvement in appearance from the initial condition but not completely optimal for this subject; touch‐up would slightly improve the result); 5, very much improved (optimal aesthetic result for the implant in this subject)] [13].

### 2.5 Data Analysis

#### 2.5.1 Baseline Characteristics

Descriptive statistics were calculated at the group level for baseline characteristics [sex, age, ethnicity, BMI, dominant hand, dominant chewing side, and dominant zygomatic arch side (that with greater anatomical or 3D spatial predominance)].

#### 2.5.2 Imaging Data

The Mirror Software Suite was used to automatically align the pretreatment images with the 3D images captured by the Vectra H2 system during the first month of follow‐up to ensure the accuracy of subsequent analyses. The integrity and consistency of the data were checked, and missing values and outliers were handled. The Mirror Software Suite was used to calculate local volume changes (in cubic centimeters) and vector skin displacement (differences between baseline and follow‐up images) for each injected facial region, with standard deviations (SDs). Between‐group differences in volume changes in different facial regions were analyzed.

#### 2.5.3 GAIS Scores

Group mean GAIS scores with SDs were calculated. The characteristics of GAIS scores for the Vectra H2 images in the two groups were described. GAIS scores for the three image types were compared.

All statistical analyses were performed using Python software (SciPy 1.8.0, statsmodels 0.13.2). The significance level was set to *p* < 0.05.

## 3 Results

### 3.1 Baseline Characteristics

In group A, the average age was 38.00 ± 6.29 years and the average BMI was 21.09 ± 1.75 kg/m^2^. Four patients preferred to chew on the left side and six patients preferred to chew on the right side. Eight patients had dominant left zygomatic arches and two patients had no dominant zygomatic arch. In group B, the average age was 41.50 ± 6.72 years and the average BMI was 20.21 ± 1.17 kg/m^2^. Five patients each preferred to chew on the left and right sides. Six patients had dominant left zygomatic arches, three patients had dominant right arches, and one patient had no dominant zygomatic arch. All patients in the sample were right handed (Table 1). No relevant adverse events were observed during the course of this study.

**TABLE 1:** Demographic data and patient medical history information of the patients in Group A and Group B included in this observational analysis.

	Group A	Group B	Mean difference [95% CI]	Statistical test	*p*	Effect size
Age (mean ± SD)	38.00 ± 6.29	41.50 ± 6.72	−2.4 [−9.5, 4.7]	*t*(18) = −0.709^a^	0.488	*d* = −0.32
BMI (mean ± SD)	21.09 ± 1.75	20.21 ± 1.17	0.64 [−0.78, 2.07]	*t*(18) = 0.949^a^	0.355	*d* = 0.42
Dominant hand	Left (*n* = 0, 0%); right (*n* = 10, 100%)	Left (*n* = 0, 0%) right (*n* = 10, 100%)				
Dominant chewing side	Left (*n* = 4, 40%); right (*n* = 6, 60%)	Left (*n* = 5, 50%); right (*n* = 5, 50%)				
Dominant zygomatic arch side	Left (*n* = 8, 80%); right (*n* = 0, 0%); none (*n* = 2, 20%)	Left (*n* = 6, 60%); right (*n* = 3, 30%); none (*n* = 1, 10%)				

*Note:* Values are presented as mean ± SD. Effect size: Cohen's *d*.

Abbreviation: CI, confidence interval.

^a^ Independent samples *t*‐test used after confirming assumptions (Shapiro–Wilk test for normality: all *p* > 0.05; Levene's test for homogeneity of variance: all *p* > 0.05).

### 3.2 Volume Changes

The postoperative superolateral facial volumes were generally increased from baseline in both groups. The average increase in group A was 0.46 ± 0.36 cm^3^ (right, 0.50 ± 0.35 cm^3^; left, 0.42 ± 0.38 cm^3^) and that in group B was 0.23 ± 0.38 cm^3^ (right, 0.26 ± 0.47 cm^3^; left, 0.19 ± 0.51 cm^3^), with no significant difference between groups (*p* = 0.067).

For the inferolateral face, the average change in group A was 1.16 ± 1.03 cm^3^ (right, 1.06 ± 0.89 cm^3^; left, 1.26 ± 1.12 cm^3^) and that in group B was −0.76 ± 0.96 cm^3^ (right, −0.65 ± 1.09 cm^3^; left, −0.87 ± 0.86 cm^3^; *p* < 0.05). For the medial face, the average change in group A was 0.03 ± 0.55 cm^3^ (right, 0.09 ± 0.52 cm^3^; left, −0.03 ± 0.60 cm^3^) and that in group B was −0.37 ± 0.60 cm^3^ (right, −0.41 ± 0.80 cm^3^; left, −0.33 ± 0.34 cm^3^; *p* < 0.05; Table 2).

**TABLE 2:** Changes in average volume in different regions of the face.

Region	The average volume change (mean ± SD)		Mean difference [95% CI]	*t*(18)	*p*	Cohen's *d*	Effect size interpretation
	Group A	Group B					
Superolateral face	0.46 ± 0.36	0.23 ± 0.38	0.24 [−0.15, 0.64]	1.316	0.067	0.589	Medium
Inferolateral face	1.16 ± 1.03	−0.76 ± 0.96	1.72 [0.79, 2.65]**	3.847	0.003**	1.72	Large
Medial face	0.03 ± 0.55	−0.37 ± 0.60	0.50 [−0.13, 1.13]	1.639	0.023*	0.733	Medium

*Note:* Values are volume changes in cm^3^, presented as mean ± SD. Significance levels: *p < 0.05, **p < 0.01. Cohen's *d* interpretation: Small (0.2–0.5), Medium (0.5–0.8), Large (> 0.8).

Abbreviation: CI, 95% confidence interval.

### 3.3 Regional Skin Vector Displacement

**FIGURE 4:**
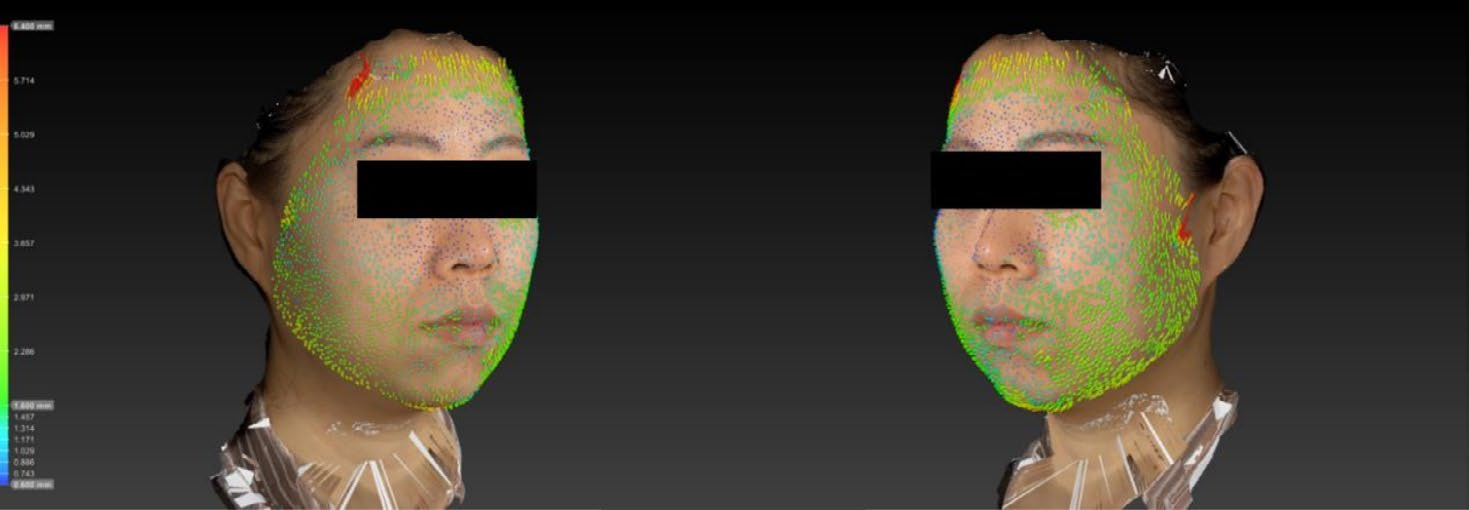
3D image of a female patient posttreatment, with superimposed color‐coded skin vector displacement mapping illustrating skin movement. Notably, the displacement vectors exhibit directional orientation toward the temporal region.

**FIGURE 5:**
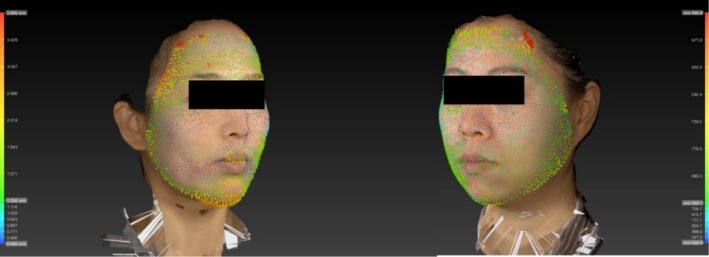
3D images of female patients treated under Group A (left) and Group B (right), with superimposed color‐coded skin vector displacement mapping illustrating skin movement.

Significant postoperative skin vector displacement was observed in both groups. In multiple patients, the magnitude of displacement declined markedly upon the crossing of the surface projection of the ligament line (i.e., it was smaller in the inferolateral and medial regions than in the superolateral region; Figure 4). Group B demonstrated generally less attenuation than group A, particularly in the inferolateral region (Figure 5).

### 3.4 GAIS Scores

The mean GAIS score was significantly lower in group A than in group B with the inclusion (2.90 ± 0.32 vs. 3.50 ± 0.53, *p* < 0.05) and exclusion (2.97 ± 0.42 vs. 3.40 ± 0.35, *p* < 0.05) of patient self‐assessments (Table 3). For the total sample, the mean GAIS score for the two‐dimensional (2D) digital images (2.73 ± 0.29) was significantly lower than those for the Mirage P2 (3.28 ± 0.42, *p* < 0.001) and Vectra H2 (3.18 ± 0.48, *p* = 0.001) images, with no significant difference between the latter (*p* = 0.621; Table 4).

**TABLE 3:** Global Aesthetic Improvement Scale (GAIS).

Statistical parameters	Group A (*n* = 10)	Group B (*n* = 10)	Statistical test	Test statistic	*p*	Effect size	95% CI
Excluding self‐assessments							
Mean ± SD	2.97 ± 0.42	3.40 ± 0.35	—	—	—	—	[2.77–3.17] vs. [3.23–3.57]
Assumptions testing							
Normality test	—	—	Shapiro–Wilk (Group A)	*W* = 0.926	0.404	—	—
	—	—	Shapiro–Wilk (Group B)	*W* = 0.918	0.336	—	—
Homogeneity of variance	—	—	Levene's test	*F* = 0.68	0.421	—	—
Primary analysis							
Parametric test	—	—	Independent *t*‐test	*t* = −2.49	0.023*	Cohen's *d* = 1.11	[0.07–0.79]
Nonparametric test (validation)	—	—	Mann–Whitney *U*	*U* = 24.5	0.029*	*r* = 0.49	—
Including self‐assessments							
Mean ± SD	2.90 ± 0.32	3.50 ± 0.53	—	—	—	—	[2.74–3.06] vs. [3.24–3.76]
Assumptions testing							
Normality test	—	—	Shapiro–Wilk (Group A)	*W* = 0.825	0.027*	—	—
	—	—	Shapiro–Wilk (Group B)	*W* = 0.901	0.221	—	—
Homogeneity of variance	—	—	Levene's test	*F* = 2.84	0.109	—	—
Primary analysis							
Nonparametric method (primary)	—	—	Mann–Whitney *U*	*U* = 18.0	0.009**	*r* = 0.58	—
Parametric method (reference)	—	—	Independent *t*‐test	*t* = −3.12	0.006**	Cohen's *d* = 1.39	[0.20–1.00]

*Note:* Values are presented as mean ± SD. Significance levels: *p < 0.05, **p < 0.01.

Abbreviation: CI, confidence interval.

**TABLE 4:** The Global Aesthetic Improvement Scale (GAIS) of the images captured by three kinds of imaging devices.

Imaging device type	*n*	Mean ± SD	95% CI	Minimum–maximum	Shapiro–Wilk	Mauchly's test	Nonparametric method (primary)	Parametric method (reference)	Post hoc pairwise comparisons (Wilcoxon signed‐rank test, Bonferroni correction)
VECTRA H2	20	3.18 ± 0.48	[2.96–3.41]	2.33–4.00	*W* = 0.936, *p* = 0.209, Normal Distribution	*W* = 0.892, *p* = 0.326, Assumption Satisfied	Friedman Test: *χ* ^2^(2) = 16.84, *p* < 0.001***, Kendall's *W* = 0.421, Medium Effect	Repeated‐measures ANOVA: *F*(2, 38) = 12.36, *p* < 0.001***, $\eta_{p}^{2}$ = 0.394, Large Effect	MIRAGE P2 vs. VECTRA H2: Mean Difference ± SE = 0.10 ± 0.06, *Z* = −1.26, Raw *p* = 0.207, Bonferroni‐Corrected *p* = 0.621, Effect Size *r* = 0.2, 95% CI for Difference = [−0.02, 0.22], Cohen's *d* = 0.22 MIRAGE P2 vs. Nikon D750: Mean Difference ± SE = 0.55 ± 0.09, *Z* = −3.78, Raw *p* < 0.001, Bonferroni‐Corrected *p* < 0.001***, Effect Size *r* = 0.6, 95% CI for Difference = [0.37, 0.73], Cohen's *d* = 1.47 VECTRA H2 vs. Nikon D750: Mean Difference ± SE = 0.45 ± 0.08, *Z* = −3.52, Raw *p* < 0.001, Bonferroni‐Corrected *p* = 0.001**, Effect size *r* = 0.56, 95% CI for Difference = [0.29, 0.61], Cohen's *d* = 1.13
MIRAGE P2	20	3.28 ± 0.42	[3.09–3.48]	2.67–4.00	*W* = 0.912, *p* = 0.072, Normal Distribution				
Nikon D750	20	2.73 ± 0.29	[2.60–2.87]	2.33–3.33	*W* = 0.878, *p* = 0.017*, Violation of Normality				

*Note:* Values are presented as mean ± SD. General *p*‐value notations: *p < 0.05, **p < 0.01, ***p < 0.001. Bonferroni correction: Due to multiple pairwise comparisons, the adjusted significance level *α* = 0.05/3 = 0.017. Under this correction, the ** and *** notations retain their original implications of higher significance levels (*p* < 0.01 and *p* < 0.001 respectively).

## 4 Discussion

In this study, a dual subjective and objective assessment approach was employed to compare the effects of two protocols for the injection of equivalent filler volumes into the lateral face. The investigation focused on three key parameters: regional volume changes, skin vector displacement, and subjective aesthetic outcomes. Both study groups consisted exclusively of female patients, with no significant difference in age or BMI. Among the 20 participants, 70% (*n* = 14) had dominant left zygomatic arches, 15% (*n* = 3) had dominant right zygomatic arches, and 15% (*n* = 3) had no dominant zygomatic arch. Despite the limited sample size, these findings suggest that left‐side zygomatic arch dominance is a prevalent craniofacial characteristic in the female Han Chinese population in Sichuan Province. Clinically, this anatomical asymmetry significantly influenced therapeutic outcomes; the vector displacement analysis revealed consistently enhanced midface lifting efficacy on the dominant zygomatic arch side with the injection of an identical filler volume.

The biomechanical advantages on the dominant zygomatic arch side may manifest through two mechanisms: tissue tension dynamics and leverage optimization. The results of our vector displacement may suggest the correlation of pronounced lateral expansion with reduced tissue extensibility in the medial face (maxillary and medial mandibular regions) and inferolateral face (lateral mandibular region), resulting in greater displacement magnitudes under equivalent injection forces. Accordingly, we may hypothesize that the elevation of the zygomatic arch extended the force arm while shortening the resistance arm according to the lever principle, thereby enhancing mechanical efficiency during the lifting procedures. These observed biomechanical patterns align with fundamental principles of facial soft tissue dynamics, whereby skeletal prominence directly influences overlying tissue tension and displacement characteristics.

The maximum posttreatment skin vector displacement was observed in the superolateral face (temporal region), with significant attenuation observed adjacent to the cutaneous projection of the zygomatic ligament. Mechanistically, this true ligament—a dense connective‐tissue structure extending from the periosteum to the dermis—functions as a biomechanical “anchor” that not only restricts soft tissue descent but also limits therapeutic lifting efficacy. The antagonistic movement of the midfacial skin across ligamentous boundaries has been documented previously [7].

Our comparison of treatment outcomes in the two groups of patients revealed that the distribution of part of the same volume of filler to the supraperiosteal layer of the zygomatic arch (i.e., the group B treatment) achieves a better facial lifting effect. That is, the lifting protocol of injecting 0.7 cm^3^ into the superficial layer of the deep temporal fascia in the posterosuperior temporal region and, simultaneously, 0.3 cm^3^ into the supraperiosteal layer of the zygomatic arch is superior to the lifting protocol of injecting 1.0 cm^3^ into the superficial layer of the deep temporal fascia in the posterosuperior temporal region. This phenomenon may be attributed to the volumetric expansion generated by supraperiosteal injection along the zygomatic arch, which exerts cephalad‐directed force on the zygomatic ligament. This mechanical action directly elevates the superficially positioned tissues of the medial and inferolateral face, thereby diminishing the ligament's restrictive effect on posterotemporal lifting. This mechanism can be further interpreted as introducing an additional lifting vector deep to the zygomatic ligament in the supraperiosteal plane. When combined with the traction force of temporal lifting, it generates biomechanical synergy through vector summation, thereby enhancing the midfacial elevation efficacy. Certainly, the above explanations require verification through quantitative analytical studies with larger sample sizes.

A methodological strength of this study is the standardized use of the identical filler type and volume in both injection protocols. This controlled approach isolated the impacts of the injection techniques, enabling the direct comparison of their efficacy. In the first protocol, all 1.0 cm^3^ of the filler was distributed to the superficial layer of the deep temporal fascia in the hair‐bearing region of the posterosuperior temporal region. In the second protocol, the amount of filler distributed to this site was reduced, and 0.3 cm^3^ filler was injected into the supraperiosteal layer of the zygomatic arch, resulting in a better facial lifting effect. To a certain extent, the study results indicate that the use of the posterotemporal lifting technique with multipoint injection into the supraperiosteal layer of the zygomatic arch achieves a better lifting effect than does the simple temporal lifting technique.

Researchers have expressed a “less is more” perspective regarding the temporal lifting technique [9]. We hypothesize that dense connective tissues (e.g., retaining ligaments) along the lifting trajectory impose biomechanical thresholds on vertical soft tissue mobilization. Once the critical load‐bearing limit has been reached, the injection of additional filler generates lifting forces that cannot be transmitted effectively beyond ligamentous anchoring points exhibiting maximal elastic deformation, resulting in the failure to enhance lower facial repositioning. This hypothesis parallels surgical principles observed during the performance of facelift procedures, with superficial fatty layer dissection beyond the ligamentous line—particularly at McGregor's patch—being required to achieve sustained elevation of the nasolabial fat pad [12].

On this basis, we propose a “combined vectors, more effectors” hypothesis; that is, when using the temporal lifting technique for facial rejuvenation, supporting the bases of inherently restrictive tissues such as ligaments along the lifting route may generate better combined lifting efficiency. The lifting force generated by filler expansion in the superficial layer of the deep temporal fascia in the posterosuperior temporal region could face counterforces from the gravity of the underlying soft tissues and the elastic recoil of ligaments along the lifting path. The lifting force generated by filler expansion in the supraperiosteal layer of the zygomatic arch in the subligamentous region may partially counteract gravitational loading on underlying soft tissues, and the volumetric support formed in this region could restrict the elastic recoil of the superiorly located ligament. From another perspective, this cephalad‐directed lifting force might confront downward pressure arising from the recoil of the ligament, and the force produced in the posterosuperior temporal region may mitigate the impact of this downward pressure on the lifting efficacy of inferiorly placed filler by restricting ligamentous recoil. Through this dual‐action mechanism, the combined protocol examined in this study is likely to reduce counteracting forces while amplifying effective lifting vectors, ultimately potentially producing a synergistic effect exceeding the cumulative efficacy of separately applied individual interventions.

There is much controversy among clinicians regarding whether filler injection only in the ligament region produces a lifting effect. We believe that volume expansion inevitably has such an effect. Filler injection into all high‐position regions, with and without obvious ligaments, and into the deep or superficial layer, will have a lifting effect on the lower‐position soft tissues as long as it results in volume expansion. In addition to the lifting effect, single‐point injection with a blunt cannula into the superficial layer of the deep temporal fascia in the posterosuperior temporal region produces local volume changes in different facial regions.

Following injection, patients in both groups in this study exhibited corresponding volume changes in the temporal region (superolateral face), lateral mandibular region (inferolateral face), and medial face. Notably, both groups showed a trend of volume augmentation in the superolateral face. This phenomenon is reasonable, as filler injected in this region compresses adjacent soft tissues, causing mild elevation in non–hair‐bearing areas. The increase in temporal volume was greater in group A (1.0 cm^3^ filler, 0.46 ± 0.36 cm^3^) than in group B (0.7 cm^3^ filler; 0.23 ± 0.38 cm^3^), and some patients in group A exhibited more noticeable temporal region bulging than did those in group B. These findings suggest that appropriate posterotemporal filler placement effectively addresses temporal hollowing. When combining temporal lifting with hollow augmentation, the performance of lifting first enables precise filler dosage assessment to prevent overfilling.

Notably, the co‐occurrence of volume reduction in the medial/inferolateral face and improved GAIS scores may not be contradictory, but rather two distinct phenomena potentially driven by a common core mechanism: oblique soft tissue stretch. Both Group A and Group B likely exhibited this oblique stretch effect, though differences in injection protocols might have led to variations in stretch force intensity. The volume reduction in the medial/inferolateral face may primarily reflect the macroscopic effect of this stretch force: under oblique traction, a portion of the soft tissue in these two regions might have migrated outward to the superolateral (temporal) and zygomatic regions, leading to a measurable decrease in local tissue volume. Given that GAIS primarily assesses facial contour tightness and sagging correction, this intra‐regional reorganization might directly address key aesthetic concerns targeted by the intervention, which could contribute to the observed score improvement. Despite subtle variations in expression during imaging, group B showed greater soft tissue improvement than did group A.

This study also involved the subjective aesthetic evaluation of images obtained with three different devices, and comparison thereof. Two devices generated 3D outputs through multi‐angle synthesis, and one (the digital camera) generated 2D images from multiple angles. Ratings were better for the synthesized 3D images than for the 2D images, potentially because the 3D images can more intuitively display the contour changes resulting from treatment, closer to the real‐world appearance. However, several limitations of this comparative assessment must be acknowledged. First, observer bias could not be eliminated fully despite the use of standardized evaluation protocols. Although all observers received training in GAIS scoring, their subjective interpretations of aesthetic improvements may have been influenced by the differences in visual fidelity and interactivity between the 2D and 3D images (e.g., 3D rotational viewing may have amplified perceived changes). Second, the learning curves associated with the use of the imaging systems were not quantified. Operators' greater familiarity with either the Vectra H2 or Mirage P2 system may have affected image acquisition quality, and subtle differences in lighting calibration or alignment precision between devices could have confounded comparison. Third, the fixed 1‐min observation time per image set may have disproportionately advantaged the 3D sets, as their interactive features add to the time required for comprehensive assessment relative to that required for static 2D image assessment. It is undeniable that the lack of an assessment of inter‐rater reliability represents one of the limitations inherent to this study.

The combined subjective aesthetic evaluation and objective measurement of facial change indicators is an advantage of this study. Technological progress has enabled the quantification of changes in various parameters resulting from facelift treatments. In addition, the zygomatic ligament was added in this study, and the lateral functional region was divided into the superolateral face (temporal region) above this ligament and the inferolateral face (lateral mandibular region) below it [7]. This division helped to clarify the lever and skin displacement restriction functions of the zygomatic arch and ligament during facial lifting.

Another advantage of this study is the adoption of a safer subcutaneous plane injection protocol. We did not use a skin entry point located 0.5–1.5 cm anterior to the tragus and vertical insertion of the blunt cannula as described previously because these parameters are relatively consistent with the location and direction of the superficial temporal artery and vein. Although these structures are in the superficial temporal fascia layer, they are separated from the subcutaneous layer into which the blunt cannula is inserted only by the fascia's thin superficial layer and could be damaged or receive injected filler during hole piercing and needle insertion [9, 14, 15]. In addition, the insertion of the blunt cannula parallel to the blood vessels is more likely to puncture the blood vessel lumen and cause vascular embolism. To minimize such iatrogenic injury, we positioned the skin entry points external to the anatomical course of these vessels and oriented the blunt cannula obliquely relative to the vascular axis, rather than parallel to it.

This study has inherent limitations, including its retrospective design, small sample, and short follow‐up period, which may have reduced the statistical power to detect subtle differences. However, the trend reflected by the results can still be taken to represent the general situation. In addition, due to practical conditions, we included only female Asian patients; anatomical differences in patients of other ethnicities and male patients may affect technique applicability.

The potential influence of ethnicity and gender on lifting efficacy may largely stem from differences in the prominence of the zygomatic arch across these demographic categories. According to our hypothesis, supporting the dense connective tissues along the lifting trajectory essentially involves artificially enhancing the protrusion of the underlying bony base. Consequently, ethnicities, genders, and individuals with inherently prominent zygomatic arch features may possess a certain degree of advantage when undergoing temporal lifting procedures. Currently, there is no consensus regarding ethnic differences in zygomatic arch characteristics. Some studies have indicated that compared to Caucasians, Asians exhibit a wider zygomatic arch and more prominent zygia—that is, the more prominent bony bases we referenced [16]. In terms of gender, research has observed that Asian females display a more prominent zygomatic arch region [16]. Naturally, these speculations await validation through further clinical statistical evidence and anatomical studies.

## 5 Conclusion

This study demonstrated that the integration of additional lifting vectors along the trajectory used with the temporal lifting technique generates enhanced synergistic lifting efficacy. In addition, the subjective evaluations of patients and observers indicated that 3D imaging provides superior representation of real‐world facial transformations, particularly subtle morphological alterations, caused by facelift treatments than does 2D imaging.

## Author Contributions

Wencheng Fan, Yanping Gao, Xiaohui Qin, Lin Chen, Li Zou, Chuanxiu Zeng, and Linghuan Zeng made substantial contributions to the conception and design of the work, data acquisition, or analysis and interpretation of data. All authors participated in drafting the manuscript or critically revising it for important intellectual content and approved the final version to be published. Each author has participated sufficiently in the work to take public responsibility for appropriate portions of the content and agrees to be accountable for all aspects of the work, ensuring that questions related to the accuracy or integrity of any part of the work are appropriately investigated and resolved.

## Ethics Statement

This study was conducted in accordance with the Declaration of Helsinki. This retrospective data analysis was conducted in compliance with regional legislation (China). Its protocol was approved by the Ethics Committee of Sichuan Integrative Medicine Hospital (no. SCZXY‐KY‐2024‐025‐M‐001). Patients who declined to grant access to their medical records were excluded from the analysis.

## Consent

As routine clinical practice, informed consent was obtained from all patients before invasive treatment. Linghuan Zeng hereby grants Journal of Cosmetic Dermatology permission to use the attached photo in the publication of the article titled “Complementary Strategies for Temporal Lifting Techniques: Biomechanical Insights from Combined Injection Techniques.”

## Conflicts of Interest

The authors declare no conflicts of interest.

## Data Availability

The data that support the findings of this study are available from the corresponding author upon reasonable request.
